# Molecular Big Data in Sports Sciences: State-of-Art and Future Prospects of OMICS-Based Sports Sciences

**DOI:** 10.3389/fmolb.2021.815410

**Published:** 2022-01-11

**Authors:** Maha Sellami, Mohamed A. Elrayess, Luca Puce, Nicola Luigi Bragazzi

**Affiliations:** ^1^ Physical Education Department, College of Education, Qatar University, Doha, Qatar; ^2^ Biomedical Research Center, Qatar University, Doha, Qatar; ^3^ QU Health, Qatar University, Doha, Qatar; ^4^ Department of Neuroscience, Rehabilitation, Ophthalmology, Genetics, Maternal and Child Health (DINOGMI), University of Genoa, Genoa, Italy; ^5^ Laboratory for Industrial and Applied Mathematics (LIAM), Department of Mathematics and Statistics, York University, Toronto, ON, Canada; ^6^ Postgraduate School of Public Health, Department of Health Sciences (DISSAL), University of Genoa, Genoa, Italy; ^7^ Section of Musculoskeletal Disease, National Institute for Health Research (NIHR) Leeds Musculoskeletal Biomedical Research Unit, Leeds Institute of Molecular Medicine, Chapel Allerton Hospital, University of Leeds, Leeds, United Kingdom

**Keywords:** molecular big data, data science, sports medicine, sports sciences, exercise and physical activity, OMICS sports sciences

## Abstract

Together with environment and experience (that is to say, diet and training), the biological and genetic make-up of an athlete plays a major role in exercise physiology. Sports genomics has shown, indeed, that some DNA single nucleotide polymorphisms (SNPs) can be associated with athlete performance and level (such as elite/world-class athletic status), having an impact on physical activity behavior, endurance, strength, power, speed, flexibility, energetic expenditure, neuromuscular coordination, metabolic and cardio-respiratory fitness, among others, as well as with psychological traits. Athletic phenotype is complex and depends on the combination of different traits and characteristics: as such, it requires a “complex science,” like that of metadata and multi-OMICS profiles. Several projects and trials (like ELITE, GAMES, Gene SMART, GENESIS, and POWERGENE) are aimed at discovering genomics-based biomarkers with an adequate predictive power. Sports genomics could enable to optimize and maximize physical performance, as well as it could predict the risk of sports-related injuries. Exercise has a profound impact on proteome too. Proteomics can assess both from a qualitative and quantitative point of view the modifications induced by training. Recently, scholars have assessed the epigenetics changes in athletes. Summarizing, the different omics specialties seem to converge in a unique approach, termed sportomics or athlomics and defined as a “holistic and top-down,” “non-hypothesis-driven research on an individual’s metabolite changes during sports and exercise” (the Athlome Project Consortium and the Santorini Declaration) Not only sportomics includes metabonomics/metabolomics, but relying on the athlete’s biological passport or profile, it would enable the systematic study of sports-induced changes and effects at any level (genome, transcriptome, proteome, etc.). However, the wealth of data is so huge and massive and heterogenous that new computational algorithms and protocols are needed, more computational power is required as well as new strategies for properly and effectively combining and integrating data.

## Sports Sciences, Exercise and Physical Activity

Sport, exercise, and physical activity represent complex, multi-factorial, non-linear activities at the intersection of biology, physiology, and psychology, resulting from the crosstalk of the biological make-up of the individual (genes, proteins, and other molecules), physiological and environmental variables, with psychological factors (such as motivation) also mattering. In the present review, we will introduce readers with state-of-arts applications of OMICS in the field of sports sciences.

Together with environment, experience, and behaviors/lifestyles (that is to say, diet and training), the biological and genetic make-up of an athlete plays a major role in exercise physiology (Bouchard et al., 2011; Guest et al., 2019; Nieman and Wentz, 2019). A genomics super-specialty called “sports genomics” has shown, indeed, that some DNA single nucleotide polymorphisms (SNPs) can be associated with athletes’ status, performance, and level (such as amateur versus elite/world-class athletic status), having an impact on a wide range of physical activity related behaviors and outcomes, including endurance, strength, power, sprint and speed, flexibility, energetic expenditure, neuromuscular coordination, metabolic and cardio-respiratory fitness, among others, as well as with mental characteristics and psychological traits (Ahmetov et al., 2016).

The so-called “athletic phenotype” is complex and depends on the combination of different traits and characteristics: as such, it requires a “complex science,” like that of metadata and multi-OMICS profiles. Several projects and trials (like ELITE, GAMES, Gene SMART, GENESIS, and POWERGENE) are aimed at discovering genomics- and post-genomics-based biomarkers with an adequate predictive power, statistical robustness, and interpretability of discoveries in terms of replicable, physiologically meaningful, and relevant patterns (Pitsiladis et al., 2016).

Sports genomics could enable to identify talents, optimize, and maximize physical performance outcomes, as well as it could predict the risk of sports-related injuries and the timing of return to sport (Tanisawa et al., 2020).

Exercise has a profound impact on proteome too (Sellami and Bragazzi, 2021). Proteomics can assess both from a qualitative and quantitative point of view the modifications induced by training. Recently, scholars have also assessed the epigenetics changes in athletes, establishing a new super-specialty termed as “sports epigenetics/epigenomics” (Zimmer et al., 2016). Other biomarkers have been identified at the level of the metabolome (Khoramipour et al., 2021), immunome (Nieman and Pence, 2020) and microbiome (Mohr et al., 2020), among others.

Summarizing, the different OMICS specialties seem to converge in a unique approach, termed sportomics or athlomics and defined as a “holistic and top-down,” “non-hypothesis-driven research on an individual’s metabolite changes during sports and exercise” (as stated in the Athlome Project Consortium and in the Santorini Declaration) (Bragazzi et al., 2020). Not only sportomics includes metabonomics/metabolomics, but relying on the athlete’s biological passport or profile, it would enable the systematic study of sports-induced changes and effects at any level (genome, transcriptome, proteome, metabolome, etc.) (Pintus et al., 2021).

These aspects will be overviewed and synthesized in the next sub-sections and paragraphs.

## Sports Genetics/Genomics

A major application of sports genetics/genomics is the dissection of the molecular basis underlying performance outcomes. As previously mentioned, various SNPs have been found to be associated with athletes’ characteristics. For example, vitamin D receptor (VDR) polymorphisms seem to be related to physical strength, bradykinin receptor B2 (BDKRB2) seems associated to strength and efficiency in muscle contraction, DRD2 to physical behavior and motivation, alpha-actinin-3 (ACTN3) to muscle contraction, speed and power, alpha-2B adrenergic receptor (ADRA2B) to metabolism, ATPase Na^+^/K^+^ transporting subunit alpha 2 (ATP1A2) with aerobic capacity, peroxisome proliferator activated receptor alpha (PPAR-alpha) with cardiorespiratory fitness and endurance, peroxisome proliferator-activated receptor delta (PPAR-delta) with aerobic capacity, and angiotensin-converting enzyme (ACE) with endurance, among others (Ahmetov and Fedotovskaya, 2015).

Ahmetov et al. (Ahmetov and Fedotovskaya, 2015) have reviewed the genetic/genomic influence on sports success achievement and performance outcomes, synthesizing and appraising currently available knowledge related to 155 genetic biomarkers (involving virtually all chromosomes and even mitochondrial DNA). In general, these markers were associated to elite athlete status, with 93 of them predicting endurance, and 62 being linked to power/strength. Forty-one biomarkers had been recently discovered, conducting genome-wide association studies (GWAS) of non-White Caucasian athletes—Afro-American, individuals from Jamaica, Russia and Japan, emphasizing the importance of investigating different ethnicities. In terms of replicability, at least two and three studies had replicated the findings related to 31 and 12 biomarkers, respectively, with 29 markers not being replicated by at least one study. This may suggest that these markers are false-positive, or that they would contribute to athlete’s status and related phenotype in some but not other ethnicities. The possibility of the influence of other factors and variables not taken into account should also not be ruled out, warranting further high-quality, multi-center research, involving large cohorts of athletes and underlining the importance of large-scale databases and Big Data.

Besides enabling discovery of the genetics underlying physical performance outcomes, sports genetics/genomics could as well predict the risk of sports-related injuries. Genes such as matrix metallopeptidase (MMP) type 3 (MMP-3), bone morphogenetic protein type 4 (BMP-4), fibroblast growth factor type 3 (FGF-3), the receptor Activator of Nuclear Factor κ B (RANK)/receptor Activator of Nuclear Factor κ B ligand (RANKL)/osteoprotegerin (OPG) signaling pathway seem to predict injury risk (Ahmetov and Fedotovskaya, 2015). Apolipoprotein E, interleukins (ILs, like IL-6 and IL-12), toll-like receptors (TLRs, such as TLR-4), nuclear factor (NF-κB), follicle stimulating hormone, chorionic gonadotropin, protein kinase catalytic subunit and solute carrier family 17 member 7 (SLC-17A7), as well as other genes encoding factors/proteins implied in the hypothalamic-pituitary-adrenal (HPA) axis seem to be associated with the prognosis after an injury (Ahmetov and Fedotovskaya, 2015).

For instance, we could consider the example of Achilles tendinopathy, which is one of the commonest sports-related injuries (Kakouris et al., 2021), with a yearly incidence of 7–9% and affecting approximately one third of runners (Ackermann and Renström, 2012). Rahim and others (Rahim et al., 2016) sampled 108 and 87 subjects with Achilles tendinopathy from South Africa and the United Kingdom, respectively. Individuals were genotyped for 5 SNPs at the level of the genes encoding the vascular endothelial growth factor (VEGF) A isoform (VEGFA) and the kinase insert-domain receptor (KDR). Polymorphisms affecting the angiogenesis signaling cascade may be involved in the etiopathogenesis of Achilles tendinopathy. Another common sports injury is given by the rotator cuff pathology (Blevins, 1997). Recent genomics studies have identified some SNPs (Cohen et al., 2020; Tashjian et al., 2021) involving the gene encoding Zinc Finger Protein 804A (ZNF804A), the glucocorticoid induced 1 gene (GLCCI1), and the gene encoding the thrombospondin type-1 domain-containing protein 7A (THSD7A) as molecular factors responsible for the development of shoulder pathologies. Some genes, such as THSD7A, were found to be downregulated, whereas other genes such as those coding the tissue inhibitor of MMP type 2 (TIMP2), Collagen Type V Alpha 1 Chain (Col5A1), Transforming Growth Factor Beta Receptor 1 (TGFBR1), and tenascin C (TNC), were detected to be upregulated.

Genetics/genomics studies have led to the design of polygenic/genotype-based scores for profiling athletes (Sillanpää et al., 2021), in terms of power, sprint, speed, and/or endurance (Ruiz et al., 2009; Ben-Zaken et al., 2015) and predict/forecast the effects of training programs and strategies (Lee et al., 2021).

Summarizing, sports genomics-related studies have covered three major and broad research areas in the field of sports and exercise sciences: namely, i) determinants of performance outcomes, ii) injury susceptibility and prevention, iii) injuries and diseases management (Griswold et al., 2021; McAuley et al., 2021).

## Sports Transcriptomics and Post-Transcriptomics/miRNAomics

MicroRNAs (miRNAs) are small single-stranded non-coding RNA molecules (generally consisting of approximately 22 nucleotides) that are involved in several cellular processes, including RNA silencing and post-transcriptional modulation of gene expression. Physical activity and sports exercise are known to tune and regulate genes as well as miRNAs, even though the expression of human miRNAome as response and adaptation to exercise/training remains to be elucidated. A recent study (Hecksteden et al., 2016) found that, in a sample of endurance and strength athletes, some miRNAs were differentially expressed in blood and plasma before and after exercise, in particular, miR-140-3p, which is involved in the pathogenesis of several malignancies (Kapodistrias et al., 2017). Other miRNAs were miR-140-5p and miR-650, the function of which is related to heart physiology (Hecksteden et al., 2016), and other molecules involved in the tuning of signaling cascades such as the insulin-like growth factor 1 (IGF-1)/phosphoinositide 3-kinase/phosphatidylinositol 3-kinase (PI3K)/protein kinase B (AKT)/mammalian (or mechanistic) target of rapamycin (mTOR) pathway (Domańska-Senderowska et al., 2019). These miRNAs have been found to correlate with some physiological parameters, such as VO_2_ max, response to training load, and modulation of skeletal muscles (Domańska-Senderowska et al., 2019). These miRNAs are muscle-specific microRNAs and are crucial in skeletal muscle development: as such, they are called myomiRNAs (Horak et al., 2016) and can be found encapsulated in muscle-derived exosomes (Mytidou et al., 2021), which are fundamental for local tissue communication and interactions (McCarthy, 2011). A recent exploration found out that also urine myomiRNAs containing exosomes can be useful for sports performance monitoring (Kuji et al., 2021).

Transcriptomic profiling studies have identified unique signatures as adaption/response to exercise, consisting of various transcription factors (most of which are belonging to the activating transcription factor (ATF)/cyclic AMP-responsive element-binding (CREB)/activator protein type 1 (AP-1) superfamily) and co-activators (Fisher et al., 2017; Pillon et al., 2020; Makhnovskii et al., 2021).

Long non-coding RNAs (lncRNAs) (Sun et al., 2017; Yao et al., 2019) are emerging as novel biomarkers of response and adaption to exercise and training (De Sanctis et al., 2021). lncRNAs are molecules that do not have any protein-coding potential but are considered to be major key players in gene expression networks by finely tuning nuclear architecture as well as transcription in the nucleus and controlling and preserving mRNA stability. lncRNAs are involved as well in other cellular and biological processes, including translation and post-translational modifications at the level of the cytoplasm (Sun et al., 2017; Yao et al., 2019).

Summarizing, complex networks consisting of interactions between a wide range of molecules—including mRNAs, miRNAs, and non-coding RNAs such as lncRNAs—are being dissected as important molecular mechanisms underlying physiological performance outcomes (De Sanctis et al., 2021), even though this research field is still young and warrants further exploration.

These networks can represent potential druggable targets for cardiovascular diseases (Correia et al., 2021), obesity and other metabolic impairments (Banitalebi et al., 2021), which impose a dramatically high, economic, and societal burden (Dai et al., 2020a; Dai et al., 2020b; Dai et al., 2020c; Dai et al., 2021a; Dai et al., 2021b; Bragazzi et al., 2021).

## Sports Epigenetics/Epigenomics

Epigenetics represents a bridge between the individual genetic make-up of the individual and the external surrounding environment. Epigenetics biomarkers have been recently studied as predictors/determinants of the response/adaptation to exercise and training (Seaborne and Sharples, 2020). Jacques and others (Jacques et al., 2019) performed a systematic literature review aimed to identify changes at the level of the epigenome detected in skeletal muscle after exercise training among healthy populations and found twenty-two studies, exploring a wide range of epigenetics markers, including DNA methylation and histone modifications.

Tarnowski et al. (Tarnowski et al., 2021) have reported epigenetics changes at the level of inflammatory response after exercise, while Denham and others (Denham et al., 2016) conducted a clinical trial and assessed the epigenetics modifications in a sample of eight young (aged 21.1 ± 2.2 years) men after 8 weeks of a program of resistance exercise training. Authors reported changes in methylation patterns of genes related to cellular and biological processes such as axon guidance, diabetes and metabolic impairments, and immunity as well as CpG islands, in more detail, growth factor genes—growth-hormone releasing hormone (GHRH) and fibroblast growth factor type 1 (FGF-1). These modifications were studied at the level of leukocytes (Sellami et al., 2021a). Kashimoto and coworkers (Kashimoto et al., 2016) in a mouse model found that physical activity exerts a significant impact on DNA methylation patterns at the level of brain structures, like hypothalamus, hippocampus, and cortex, emphasizing the role of exercise and physical activity as epigenetics regulators of brain plasticity and cognitive processes (Fernandes et al., 2017) and interventional measures counteracting ageing and neurodegeneration (Xu et al., 2021).

Concerning unhealthy populations, Nguyen and others (Nguyen et al., 2016) found epigenetic changes at the level of glutathione peroxidase 1 gene in a severely dyslipidemic mouse model, suggesting potential pharmacological pathways and druggable targets.

Interestingly, Grzywacz and others (Grzywacz et al., 2021) explored the correlation between DNA methylation and personality characteristics in a sample of 100 European sports male individuals aged 22.88 ± 6.35 years, age- and gender-matched with 239 healthy controls. Authors found associations between methylation patterns of the promoter sequence of the dopamine transporter gene (DAT-1) and some personality traits/features.

However, the causality and the replicability of such markers are still unknown, warranting further research in the field (Akberdin et al., 2021).

## Sports Proteomics and Metabolomics/Metabonomics

Exercise has a profound impact on proteome, finely tuning energy metabolism cascades, adenosine triphosphate (ATP pathways), and mitochondrial proteins synthesis, among others (Balfoussia et al., 2014; Hargreaves and Spriet, 2020). For instance, plasma proteome has been shown to correlate to the extreme level of physical stress experienced by athletes taking part into the Spartathlon race who exhibited differentially expressed proteins belonging to several cascades and networks, including inflammation, anti-oxidation, anti-coagulation and iron and vitamin D transport pathways (Balfoussia et al., 2014). Running as well as other exercises and physical activities may induce changes in the acetylome (Philp et al., 2014). Other biomarkers of cellular stress are given by biological processes and events such as protein sumoylation: conducting intense resistance exercises can result into an acute, transient nuclear translocation of Small Ubiquitin-Related Modifier (SUMO)-1 at the level of Human Myofibres types I and II (Gehlert et al., 2016). Proteomics can assess both from a qualitative and quantitative point of view the modifications induced by long-term moderate training, as well as the effects of acute swimming exercise and other sports disciplines (Ubaida-Mohien et al., 2019; Gomes et al., 2020). Magherini and collaborators (Magherini et al., 2013) explored protein expression and patterns and markers of oxidative stress in the *Extensor Digitorum Longus* and the Soleus. Authors found that the protein carbonylation and lipid peroxidation levels were reduced following acute swimming bursts. Also one-half minute of maximal exercise can induce changes in human urinary proteome, some of which are still visible after 2 hours, whilst prolonged strenuous exercise can cause proteinuria (haemoglobinuria or myoglobinuria), which reflects in larger proteomics alterations and biochemical/biophysical anomalies (Bellinghieri et al., 2008; Kohler et al., 2015).

Concerning sports injuries, Sejersen and others (Sejersen et al., 2015) reviewed the proteomics of rotator cuff pathologies and tendinopathies and found an increase of collagen I and III, several MMPs, including MMP-1, MMP-9, MMP-13, tissue inhibitor of MMP type 1 (TIMP-1), and VEGF, and a decrease in MMP-3. Daisy et al. (Daisy et al., 2021) recruited 47 concussion athletes from both contact and non-contact sports disciplines, who were age- and sex-matched with 48 controls. Authors identified IGF-1 and IGF binding protein 5 (IGFBP5) as urine proteomics biomarkers, highly correlating with single-task gait velocity and predictive of concussions at acute time-points.

Finally, Khoramipour and others (Khoramipour et al., 2021) were able to identify five major categories covered by sports and exercise metabolomics/metabonomics studies: namely, i) exercise metabolism; ii) exercise metabolism with focus on nutritional aspects; iii) sports metabolism; iv) exercise metabolism with an emphasis on clinical implications; and v) metabolome assessments and comparisons. Authors found that exercise metabolism was the research area most addressed by scholarly investigations, generally utilizing blood and urine samples but relying on untargeted, qualitative rather than targeted, quantitative platforms. Despite attracting increasing interest from the scientific community which reflects into a higher publishing trend, sports metabolomics/metabonomics still represents more an art than a science, warranting more high-quality studies, with samples drawn from larger cohorts and exploiting a longitudinal, randomized design.

## Emerging Molecular Sports Big Data: Sports Nutrigenomics, Immunomics and Microbiomics

Sports nutrigenomics is a young super-specialty, relatively under-explored in the existing scholarly literature (Bragazzi, 2013). It can potentially help i) devise and deliver target nutritional advice, ii) stratify and identify athletes’ metabotypes (Hillesheim et al., 2020). Few studies have attempted exploring this topic. Among the early pioneering contributions, we have to mention the study by Ribeiro et al. (Ribeiro et al., 2013), who explored the effects of erythropoietin (EPO T→G) and α-actinin-3 (ACTN3 R577X) SNPs on the response to the dietary ingestion of antioxidant supplementation based on pequi oil (*Caryocar brasiliense* Camb.) in a sample of runners. CYP1A2 genotype was found to modify the effects of caffeine uptake versus placebo on muscle strength in a sample of competitive male athletes (Wong et al., 2021).

The term “immunome” has been coined to indicate the incredibly diverse and vast individual’s repertoire of antibodies, B cell receptors and T cell receptors (TRs), that can be generally assessed from the blood, and consists of approximately 50,000–440,000 B lymphocytes and 600,000–3,500,000 T lymphocytes per ml in apparently healthy adults (Arnaout et al., 2021). Exercise can be conceived as a “molecular choreography,” that is to say, as a concerted series of biological, and cellular processes at the level of the cardiovascular, endocrine, and immunologic systems (Contrepois et al., 2020). Physical activity and sport are able to modulate the expression, production and release of cytokines, known as exerkines (Eaton et al., 2018; Sellami et al., 2021b; Magliulo et al., 2021).

Finally, sports microbiomics is a very recent and young super-specialty stemming from post-genomics specializations (Hughes, 2020; Marttinen et al., 2020). Athletes’ gut microbiome has been found to modulate the effects of nutrients uptake and dietary components and/or supplements, like probiotics, impacting as well on performance outcomes.

## Conclusion: Towards Sports Multi-OMICS

As previously mentioned, athletic phenotype is extremely complex and depends on the combination of a wide range of traits and characteristics: from genetic/genomics to post-genomics features, affecting the endocrine, immunologic systems. As such, it requires a “complex science,” like that of metadata and multi-OMICS profiles. Several ambitious multi-center trials and projects (like the previously mentioned ELITE, GAMES, Gene SMART, GENESIS, and POWERGENE) are aimed at discovering genomics-based biomarkers with an adequate predictive power.

Sportomics strategies have been so far applied to the investigation of effects of arginine, keto analogues, and other amino acids supplementation, or the determinants of performance outcomes in world-class canoeists or elite athletes (Gonçalves et al., 2012; Camerino et al., 2016; Coelho et al., 2016; Marttinen et al., 2020; Monnerat et al., 2020; Clauss et al., 2021).

The unique combination and convergence of Big Data derived from molecular techniques (OMICS), and data science offers unprecedented solutions to old problems as well as new opportunities The usage of more than one source/channel of data enables to have more realistic insights, given the complexity underlying physical performance, its drivers/predictors and its outcomes. Molecular Big Data are promising in that they are anticipated to enable a better understanding of the determinants of physical performance, shedding light on how optimizing and enhancing performance, identifying talents, predicting (nowcasting/forecasting) physiological outcomes, predicting injuries and identifying strategies for injury prevention, among others. However, the wealth of data is so huge and massive and heterogenous that new computational algorithms and protocols are needed, more computational power is required as well as new strategies for properly and effectively combining and integrating data.
